# Supplementary material for: CXCL14 as a Key Regulator of Neuronal Development: Insights from Its Receptor and Multi-Omics Analysis
Source: Int J Mol Sci. 2024 Jan 29;25(3):1651. doi: 10.3390/ijms25031651 (PMC10855946; doi:10.3390/ijms25031651)
Supplement: Supplementary file 1 [file ijms-25-01651-s001.zip › ijms-2813741-supplement/Table s1-s2.pdf]

**Table S1.** Birth rate and fertility of CXCL14-/-

| | (+/-) | (-/-) | Total |
|---|---|---|---|
| **Male** | 21 | 7 | 28(47.5%) |
| **Female** | 21 | 10 | 31(52.5%) |
| **Total** | 42(71.2%) | 17(28.8%) | 59 |

The genotype proportion of offspring born by crossbreeding of 8 week-old male CXCL14$^{-/-}$ and female CXCL14$^{+/-}$ mice, produced a total of 59 mice, 42 mice of CXCL14$^{+/-}$, and 17 mice of CXCL14$^{-/-}$, the birth rate of CXCL14$^{-/-}$ was 28.8% which was significantly different from expected percentage of 50% (n=59, $\chi^2$=10.59 p<0.01). The genotype proportion of offspring had no significant difference between males and females ($\chi^2$=0.378, p=0.539).

**Table S2.** Parturition of pregnant (-/-) females

| Failure | Dead pups | Success | Total |
|---|---|---|---|
| 1 | 7 | 2 | 10 |

Parturition of pregnant (-/-) females was observed through the crossbreeding of male CXCL14$^{-/-}$ and female CXCL14$^{-/-}$ mice in 10 cages, only three in two cages mice were born. Of particular note was that, pregnant features and abdominal bulge could be observed in CXCL14$^{-/-}$ female mice at 10-15 days of mating, then the abdomen gradually becomes flat and loses the characteristics of pregnancy after 10-15 days of mating, suggesting that the female mice may slip during this period.
